# Evaluation and Comparison of Three Common Methods for PFAS Extraction from Soybean Tissues

**DOI:** 10.1021/acsagscitech.5c00518

**Published:** 2025-11-13

**Authors:** Madhav Kharel, Yuwei Zuo, Weilan Zhang

**Affiliations:** Department of Environmental & Sustainable Engineering, 1084 University at Albany State University of New York, Albany, New York 12222, United States

**Keywords:** extraction efficiency, internal standard, PFAS, plant uptake, recovery

## Abstract

This study evaluated three methods, methyl *tert*-butyl ether-sodium hydroxide (MTBE-NaOH) method, EPA method 1633, and U.S. Food and Drug Administration (FDA) chemical analytical manual (CAM) method C-010.03, for their effectiveness in extracting PFAS from soybean tissues. EPA method 1633 consistently delivered the highest and most reproducible EIS recoveries when plant tissues contained PFAS at low levels. Regarding target PFAS extraction efficiency, EPA method 1633 also demonstrated superior performance at environmentally relevant low concentrations. At higher PFAS concentrations in plant tissues, no single method clearly dominated; however, EPA method 1633 remained consistently reliable and was never significantly outperformed by the other two methods. Overall, EPA method 1633 is recommended as the default method for routine analyses at typical environmental PFAS levels, with MTBE-NaOH method preferred when accurate isotopic correction based on EIS recovery is critical in highly contaminated plant samples. A cost comparison of these three methods further supports the preference for EPA Method 1633 and MTBE-NaOH method for plant-tissue analyses. These findings contribute to PFAS risk assessment in agricultural and food safety contexts by enhancing the understanding of PFAS interactions with edible crops.

## Graphical abstract

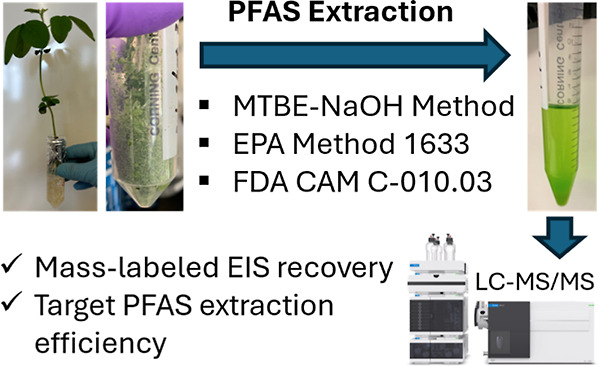

## 1 Introduction

Per- and polyfluoroalkyl substances (PFAS), extensively used in industry and consumer products for their unique properties, are increasingly detected across diverse environments.^1,2^ Growing concerns over their environmental risks have driven significant efforts to accurately identify and quantify PFAS in complex matrices. Plants are a fundamental component of the food chain and play a crucial role in trophic networks. When plants take up PFAS from contaminated water and soil, they act as a pathway for these chemicals to enter the ecosystem. Through consumption, PFAS can move up the food chain to higher trophic levels, including animals and humans.^3^ These chemicals are associated with numerous adverse health effects, such as cancer, hormone disruption, and immune system impairment.^4–10^ As a result, understanding the uptake and bioaccumulation of PFAS in plants is essential for assessing the health risks associated with consuming contaminated crops. Research on plant uptake and bioaccumulation of PFAS is extensive, addressing various factors such as bioavailability, plant physiology, and molecular responses.^11,12^ Studies have shown that the intrinsic properties of PFAS, including carbon chain length and functional groups, as well as extrinsic environmental factors, particularly soil characteristics, significantly influence the extent of accumulation in plant tissues.^11–14^ These investigations consistently depend on the quantitative analysis of PFAS in plant tissues, with PFAS extraction being a critical first step in such analyses.

Currently, various PFAS extraction methods for plant tissues are documented in the literature. We previously developed a methyl *tert*-butyl ether (MTBE)-based method for extracting PFAS from plants, which proved effective for common plant species in the northeastern United States. These species include *Juncus effusus* (soft rush),^15,16^ *Carex comosa* (longhair sedge),^17^ *Lemna minor* (duckweed),^18,19^ *Typha latifolia* (cattail),^20,21^ Glycine max (L.) Merr (soybean),^22–24^ and *Phleum pratense* (timothy-grass).^25^ In addition, basic methanol-based methods (methanol with ammonium hydroxide) have been widely utilized in PFAS-plant research.^26–28^ The U.S. EPA released method 1633A in December 2024, a standardized method for analyzing PFAS in aqueous, solid, biosolid, and tissue samples, making it one of the most commonly used protocols. However, this basic methanol-based extraction method was originally developed for PFAS in animal tissues, which lack cell walls. Although the method has demonstrated high recovery efficiencies for PFAS in fish and chicken tissues, its applicability to plant tissues with cell walls and its true extraction efficiency remain uncertain. Extraction procedures based on the quick, easy, effective, rugged, and safe (QuEChERS) method have also been frequently adopted for PFAS extraction from plants in previous studies.^29–31^ Building on these developments, the U.S. Food and Drug Administration (FDA) chemical analytical manual (CAM) method C-010.03, published on April 12, 2024, extends its application to food and feed, including plant-based samples such as lettuce, blueberries, and corn snaplage. This method employs QuEChERS salt packets and dSPE sorbents to enhance extraction efficiency, making it suitable for plant tissues. However, the use of QuEChERS kits in this method significantly increases the cost of PFAS extraction, raising concerns about its practicality for large-scale studies involving plant matrices.

Spiking extracted internal standards (EIS) at the start of the extraction process is a widely used practice in analytical chemistry to ensure accuracy, consistency, and reliability in quantifying target analytes, particularly when extracting contaminants from environmental matrices. The PFAS extraction efficiencies of the aforementioned methods are determined by quantifying isotopically labeled EIS spiked at a known mass at the beginning of the extraction procedures. This approach assumes that the EIS mimic the affinity and interactions of targeted PFAS compounds taken up and accumulated in plant tissues, enabling corrections for recovery rates and matrix effects. However, it is reasonable to question whether the binding behavior of spiked PFAS differs from that of PFAS naturally accumulated within plant tissues. As a result, the true PFAS recoveries (extraction efficiencies) achieved by these methods remain uncertain.

For this study, 10 PFAS compounds were selected as representative models based on their frequent environmental occurrence and consistent reporting in literature on plant uptake. Soybean was selected as the model plant to assess the extraction efficiencies. Three commonly used methods for PFAS extraction from plant tissues, the MTBE-NaOH method, basic methanol-based EPA method 1633, and QuEChERS-based FDA CAM C-010.03, were systematically compared. We aim to critically evaluate these methods for quantifying PFAS in plant tissues by directly comparing the extraction efficiencies of spiked EIS and those of PFAS naturally accumulated in soybean plants. This study determined whether the three methods accurately reflect the recovery of plant-incorporated PFAS, thereby providing a more reliable basis for assessing PFAS uptake and accumulation in crops. This work is essential for advancing research on PFAS uptake, accumulation, and associated agricultural and health risks. The findings also contribute to the standardization of PFAS extraction methods for plant tissues, ensuring data precision and consistency across PFAS studies.

## 2 Materials and Methods

### 2.1 Experimental Setup

The chemicals and reagents used in this study are listed in Table S1. Quarter-strength Hoagland solutions containing 10 PFAS compounds at concentrations of 2 or 200 μg/L per compound were prepared to evaluate the extraction performance of each method at low and high PFAS concentration levels in plant tissues. The lower concentration reflects typical environmental background levels relevant for routine monitoring, while the higher concentration simulates highly contaminated scenarios, allowing assessment of method reliability and accuracy under both ambient and extreme conditions. The 10 PFAS compounds included perfluorobutanoic acid (PFBA), perfluorobutanesulfonic acid (PFBS), perfluorohexanoic acid (PFHxA), hexafluoropropylene oxide dimer acid (HFPO–DA or GenX), perfluorohexanesulfonic acid (PFHxS), 6:2 fluorotelomer sulfonate (6:2 FTS), perfluorooctanoic acid (PFOA), perfluorooctanesulfonic acid (PFOS), perfluorononanoic acid (PFNA), and *N*-ethylperfluorooctane sulfonamidoacetic acid (*N*-EtFOSAA). These compounds were selected to represent a range of chain lengths, functional groups, ether-PFAS alternatives, and PFAS precursors of environmental significance. The selection aligns with regulatory monitoring priorities and includes compounds frequently detected in plants, water, and agricultural matrices.^1,2,5,12^ In addition, the studied PFAS are covered by EPA Method 537.1 ^32^ and method 8327 ^33^ for drinking water and wastewater, highlighting their relevance to environmental and human health risk assessment. The actual PFAS concentrations in nutrient solutions were measured to ensure consistency.

A total of ∼60 soybean seeds were sterilized with a 1.25% sodium hypochlorite solution for 10 min, thoroughly rinsed with deionized (DI) water, and germinated in moistened, PFAS-free Ottawa sand for 5 days. The quarter-strength Hoagland nutrient solution was prepared in deionized water using a Hoagland modified basal salt mixture (Phytotech Laboratories, Lenexa, KS) at 0.41 g/L. The ingredients of Hoagland modified basal salt mixture were shown in Table S2. The PFAS mixture was spiked to reach target concentrations of 2 μg/L and 200 μg/L. Healthy seedlings of similar size were selected and transplanted into 50 mL polypropylene centrifuge tubes containing the prepared nutrient solutions with PFAS. Twelve plant replicates were prepared for each PFAS concentration. The plants were grown in a growth cart under standard laboratory conditions (22 °C, 40–60% relative humidity) with a 16/8 h light/dark cycle provided by fluorescent lighting for 20 days. Each tube was replenished daily with freshly prepared quarter-strength Hoagland solution containing PFAS at the corresponding concentrations, and the replenished volume was recorded. A control group of soybean plants was also prepared, grown in quarter-strength Hoagland solution without PFAS exposure. After 20 days, the plants were harvested, and the roots were thoroughly rinsed with deionized water. The remaining nutrient solution from each tube within each treatment group was pooled, and the total volume and PFAS concentrations were subsequently measured. All plant tissues, including shoots and roots within the same treatment, were combined, freeze-dried at −37 °C for 24 h, weighed, and ground into powder using a coffee grinder for PFAS extraction.

### 2.2 PFAS Extraction Methods

PFAS compounds in the nutrient solution were extracted following EPA method 1633. In brief, the liquid samples were first spiked with extracted internal standards (EIS) obtained from Wellington Laboratories Inc (Guelph, Ontario, Canada) and subjected to solid-phase extraction (SPE) using Agilent Bond Elut PFAS WAX cartridges. To prevent contamination and false positives, all glassware, plasticware, and tools were rinsed with methanol and ultrapure water before use. The working area and equipment were cleaned regularly. Samples and standards were handled with PFAS-free gloves and labware, and method blanks were included in each batch to check for background contamination.

The SPE eluate was subsequently spiked with nonextracted internal standards (NIS) from Wellington Laboratories Inc. and analyzed using an Agilent 6470 triple-quadrupole LC–MS. The naming information for the related mass-labeled PFAS compounds in the EIS and NIS is shown in Table 1. The pairing of the 10 spiked PFAS compounds with their corresponding EIS and NIS, was carried out according to EPA Method 1633 and is also presented in Table 1. Details of the LC–MS/MS instrument setup, along with the limits of detection (LOD) and quantification (LOQ) and R^2^ value of the calibration curve for each PFAS compound, are provided in Table S3. An agilent eclipse plus C18 (4.6 × 50 mm, 3.5 μm) delay column was used to separate background PFAS originating from the instrument.

**Table 1:** Naming and Pairing Information of the 10 PFAS Compounds and Their Associated EIS and NIS in This Study

target PFAS	extracted internal standard (EIS)		nonextracted internal standard (NIS	
	compound name	abbreviation	compound name	abbreviation
PFBA	perfluoro-*n*-(^13^C_4_)butanoic acid	MPFBA	perfluoro-*n*-(2,3,4-^13^C_3_)butanoic acid	M3PFBA
PFBS	sodium perfluoro-1-(2,3,4-^13^C_3_)butanesulfonate	M3PFBS	sodium perfluoro-1-hexane(^18^O_2_)sulfonate	MPFHxS
PFHxA	perfluoro-*n*-(1,2,3,4,6-^13^C_5_)hexanoicacid	M5PFHxA	perfluoro-*n*-(1,2-^13^C_2_)hexanoic acid	MPFHxA
HFPO–DA	2,3,3,3- tetrafluoro-2-(1,1,2,2,3,3,3-heptafluoropropoxy (^13^C_3_)propanoic acid	M3HFPO–DA	perfluoro-*n*-(1,2-^13^C_2_)hexanoic acid	MPFHxA
PFHxS	sodium perfluoro-1-(1,2,3–13C3) hexanesulfonate	M3PFHxS	sodium perfluoro-1-hexane(^18^O_2_)sulfonate	MPFHxS
6:2FTS	sodium 1*H*,1*H*,2*H*,2*H*-perfluoro(1,2-^13^C_2_)octanesulfonate	M2–6:2 FTS	sodium perfluoro-1-hexane(^18^O_2_)sulfonate	MPFHxS
PFOA	perfluoro-*n*-(^13^C_8_)octanoic acid	M8PFOA	perfluoro-*n*-(1,2,3, 4-^13^C_4_)octanoic acid	MPFOA
PFOS	sodium perfluoro-1-(^13^C_8_) octanesulfonate	M8PFOS	sodium perfluoro-1-(1,2,3,4-^13^C_4_)octanesulfonate	MPFOS
PFNA	perfluoro-*n*-(^13^C_9_)nonanoic acid	M9PFNA	perfluoro-*n*-(1,2,3,4,5-^13^C_5_)nonanoic acid	MPFNA
*N*-EtFOSAA	*N*-ethyl-d_5_-pertiuoro-1-octanesultonamidoacetic acid	d5-*N*-EtFOSAA	sodium perfluoro-1-(1,2,3,4-^13^C_4_)octanesulfonate	MPFOS

Three extraction methods for PFAS in plant tissues were evaluated in this research: (1) MTBE-NaOH method, (2) EPA method 1633, and (3) FDA chemical analytical manual (CAM) C-010.03 method. Regarding the MTBE-NaOH method, 0.5 g of ground plant material was mixed with 4 mL of 0.4 M NaOH, spiked with EIS, and left at 4 °C overnight. The amount of EIS for spiking was calculated to ensure that the EIS concentrations in the final plant extracts fell within the calibration range and above the LOQ shown in Table S3. Subsequently, 2 mL of tetrabutylammonium hydrogensulfate (TBAHS, 0.5 M) and 4 mL of Na_2_CO_3_ buffer (0.25 M) were added. After vortexing, 5 mL of *tert*-butyl methyl ether (MTBE) was added to the mixture, which was then shaken on a nutating mixer at 60 rpm for 20 min. The organic and aqueous layers were separated by centrifugation, and the MTBE layer was transferred to a second polypropylene tube. The aqueous phase was further extracted twice with 2 × 5 mL of MTBE. All organic fractions from the three extraction rounds were combined and allowed to evaporate under nitrogen. The residue was reconstituted in 1 mL of methanol, diluted with 9 mL of water, and subjected to SPE using Agilent cartridges. The SPE eluate was spiked with NIS and analyzed for PFAS.

Regarding the EPA method, a 0.5 g sample of dried plant powder was mixed with 10 mL of 0.05 M KOH in methanol and spiked with a mixture of EIS. After vortexing, equilibrating for 30 min, and centrifuging, the liquid supernatant was collected. The extraction was repeated twice, first with acetonitrile and then with 0.05 M KOH in methanol. The supernatants from all three rounds of extraction were combined, cleaned with Envi-Carb carbon, adjusted to a pH of 6.5 ± 0.5, and diluted to 50 mL with water. Subsequently, the diluted extracts underwent SPE using the aforementioned cartridges. The SPE eluate was spiked with NIS and analyzed using triple-quadrupole LC–MS.

Following the FDA method, 0.5 g of ground plant tissue was mixed with 15 mL of LC/MS-grade water and spiked with EIS. Subsequently, 10 mL of acetonitrile and 150 μL of formic acid were added to the mixture, which was vigorously shaken for 1 min. A QuEChERS salt packet containing 6000 mg of MgSO_4_ and 1500 mg of NaCl was then added, and the mixture was vortexed until homogeneous and free of clumps. The sample was shaken on a shaker at 1500 rpm for 5 min and centrifuged at 10,000 RCF for 5 min. The resulting supernatant was transferred to a 50 mL centrifuge tube containing dSPE sorbent with 900 mg of MgSO_4_, 300 mg of PSA, and 150 mg of graphitized carbon black, then vortexed for 2 min. After another 5 min centrifugation at 1000 RCF, 5 mL of the supernatant was filtered through a 0.2 μm nylon syringe filter and transferred to a new 50 mL conical centrifuge tube. The filtrate was evaporated to approximately 1 mL under nitrogen in a 60 °C water bath, diluted with 11 mL of water, and subjected to SPE using the aforementioned cartridges. The SPE eluate was spiked with NIS and analyzed for PFAS.

For quality control (QC) purposes, method blanks, low-level ongoing precision and recovery standards (LLOPR), and midlevel ongoing precision and recovery standards (MLOPR) were prepared in each batch for all three methods. These were generated by spiking a mixture of 10 native PFAS at 2× LOQ for LLOPR and at midlevel calibration concentrations for MLOPR into dry control plant biomass without PFAS exposure, and then analyzed according to EPA method 1633.

### 2.3 Calculations of EIS Recovery and Extraction Efficiency and Statistical Analysis

The recovery of spiked EIS in all samples and spiked target PFAS in ongoing precision and recovery standards (OPR) was calculated using the following formula

$$Re=\frac{M_{recovered}}{M_{spiked}}\times 100\%$$

where: *M*_recovered_: the mass of EIS or target PFAS recovered after extraction. *M*_spiked_: the mass of EIS or target PFAS spiked before extraction.

Extraction efficiency (EE) of each targeted PFAS in plant tissue samples was calculated for each method using the following formula

$$EE=\left(\frac{M_{PFAS}\times m_{total}}{m_{extraction}}\right)/\left[\left(V_{tube}{+V}_{added}\right)\times C_{initial}-V_{final}\times C_{final}\right]\times 100\%$$

where/M_PFAS_: the PFAS mass extracted from each plant sample. *m*_extraction_: the mass of plant biomass used for extraction (0.5 g).*m*_total_: the total dry weight of the combined plant tissues for each treatment group. *V*_tube_: the total volume of nutrient solution in all 12 tubes for each treatment group before plant transfer (50 × 12 = 600 mL). *V*_added_: the total volume of nutrient solution added to all plant replicates in each treatment group during plant cultivation period. *C*_initial_: the PFAS concentration in the prepared nutrient solution for each treatment group. *V*_final_: the total combined volume of the combined remaining nutrient solution from all replicates in each treatment group after plant harvesting. *C*_final_: the PFAS concentration in the combined remaining nutrient solution.

Experimental data are presented as means ± standard deviation from three replicates. On-way and two-way analysis of variance (ANOVA) with posthoc tests were conducted using IBM SPSS Statistics 22. Statistical significance was defined as *p* ≤ 0.05.

## 3 Results and Discussion

### 3.1 Evaluation of EIS Recovery Across Three Extraction Methods

In this study, NIS were added after sample extraction but prior to instrumental analysis, primarily to account for instrument-related variability such as fluctuations in injection volume and ionization efficiency. Although NIS do not correct for analyte losses during the extraction process, they help ensure consistent calibration and enhance confidence in quantification. However, relying solely on NIS without incorporating EIS may overlook procedural losses and compromise data accuracy. For robust quality control, NIS were used in conjunction with EIS to comprehensively address both analytical and extraction-related variability (Table 1).

EIS are isotopically labeled analogs of the target PFAS compounds. Adding EIS prior to sample extraction corrects for potential losses during sample preparation and is critical for ensuring accuracy and precision in PFAS quantification. This approach is particularly important for environmental samples such as soil, biosolids, and plant tissues, where sorption and matrix interference can compromise analytical results. EPA method 1633 requires the use of EIS as standard practice for PFAS analysis and specifies acceptance limits for EIS in tissue matrices and associated QC samples (Table 2). Recovery values within the acceptable range indicate that the extraction and analytical procedures are functioning reliably. In contrast, consistently low or variable EIS recoveries may suggest poor extraction efficiency and/or instrument instability, warranting further method optimization.

**Table 2:** EIS Recoveries from Plant Tissue Samples Using the Methods Evaluated (*n* = 3) and Acceptance Limits for EIS Recoveries in Tissue Samples Specified in EPA Method 1633

method	treatment	extracted internal standard recovery (%)									
		MPFBA	M3PFBS	M5PFHxA	M3HFPO–DA	M3PFHxS	M2–6:2 FTS	M8PFOA	M8PFOS	M9PFNA	d5-*N*-EtFOSAA
MTBE-NaOH Method	0 ppb	36.09 ± 1.37	86.78 ± 2.49	36.15 ± 4.46	38.78 ± 3.20	66.98 ± 4.05	188.59 ± 15.59	72.41 ± 4.48	68.58 ± 3.83	73.89 ± 5.95	95.66 ± 3.35
	2 ppb	32.90 ± 2.19	74.86 ± 1.60	70.20 ± 1.91	52.22 ± 6.16	69.78 ± 1.20	287.12 ± 19.11	72.41 ± 1.67	73.57 ± 6.22	70.02 ± 4.82	74.36 ± 7.41
	200 ppb	45.33 ± 19.43	96.12 ± 48.82	105.36 ± 45.20	90.13 ± 42.49	98.34 ± 44.39	167.43 ± 91.76	102.97 ± 48.11	97.24 ± 26.56	103.35 ± 47.18	78.87 ± 47.44
EPA method 1633	0 ppb	65.47 ± 20.26	65.81 ± 1.32	44.64 ± 0.64	37.98 ± 2.21	55.32 ± 1.14	64.58 ± 0.48	23.68 ± 0.37	49.36 ± 3.09	27.19 ± 1.03	63.83 ± 4.86
	2 ppb	41.33 ± 7.76	60.41 ± 6.48	57.07 ± 7.19	53.89 ± 4.27	79.63 ± 2.45	148.06 ± 19.76	41.77 ± 4.26	50.91 ± 6.95	34.36 ± 3.51	85.00 ± 9.47
	200 ppb	54.26 ± 16.13	63.09 ± 6.20	62.52 ± 8.42	58.39 ± 3.37	65.32 ± 6.15	91.46 ± 9.26	59.32 ± 5.99	72.81 ± 2.38	59.89 ± 10.49	57.35 ± 13.05
FDA CAM C-010.03	0 ppb	35.01 ± 1.36	44.65 ± 2.14	32.71 ± 1.02	33.06 ± 0.49	42.98 ± 1.68	199.36 ± 8.32	39.44 ± 0.82	43.50 ± 1.51	42.42 ± 3.03	109.05 ± 4.71
	2 ppb	42.48 ± 2.31	46.03 ± 6.39	37.90 ± 2.75	37.85 ± 3.30	48.94 ± 4.96	293.22 ± 29.12	42.74 ± 1.59	46.42 ± 2.93	45.21 ± 2.62	110.92 ± 12.85
	200 ppb	37.83 ± 9.26	39.20 ± 12.16	40.11 ± 9.94	35.77 ± 12.68	45.43 ± 11.92	114.09 ± 14.81	40.95 ± 11.12	43.02 ± 20.27	43.49 ± 16.23	57.39 ± 12.43
EPA method 1633 EIS recovery acceptance limits	MPFBA	M3PFBS	M5PFHxA	M3HFPO–DA	M3PFHxS	M2–6:2FTS	M8PFOA	M8PFOS	M9PFNA	d5-N-EtFOSAA	
	5–130	25–190	25–170	20–185	35–175	35–300	25–150	40–160	35–185	30–235	

Tables 2 and S4 present the EIS recoveries from plant tissue and QC samples for each extraction method, with all values falling within the acceptable ranges specified by EPA method 1633. Two-way ANOVA was conducted to evaluate the effects of extraction method and PFAS levels in plant tissues on EIS recovery (Table S5). ANOVA revealed that the choice of extraction method strongly influenced recovery for seven of the ten EIS compounds. The exceptions were MPFBA, M3HFPO–DA, and d5-*N*-EtFOSAA, for which the three methods exhibited no statistically significant differences. The PFAS level in the plant tissues (0, 2, or 200 ppb) also affected recovery for seven EIS compounds, with no meaningful effect observed for M3PFBS, M3PFHxS, and d5-*N*-EtFOSAA. An interaction between the extraction method and PFAS level, indicating that the performance of a given method depends on the PFAS concentration in plant tissue, was detected for only two EIS compounds, MPFBA and M5PFHxA. Thus, for most EIS compounds, the performance of the three extraction methods remained consistent across PFAS levels in plant tissues.

The statistical evaluation demonstrated a significant influence of extraction method selection and PFAS levels in plant tissues on EIS recoveries. For control (0 ppb) and 2 ppb treatment, EPA method 1633 consistently produced the highest and most reproducible recoveries across the ten EIS compounds, whereas MTBE-NaOH method delivered higher average EIS recoveries for the treatment at 200 ppb, although with greater variability. The FDA method recovered less EIS across all treatment levels and never outperformed the other two methods. Taken together, the EIS recovery results support the use of EPA method 1633 for routine or low PFAS level applications and MTBE-NaOH method for plant tissues containing high PFAS levels, while discouraging exclusive reliance on the FDA method for quantitative analyses.

### 3.2 Evaluation of Extraction Efficiency Across Three Extraction Methods

The actual PFAS concentrations and masses in plant tissues were calculated from LC–MS/MS measurements, taking EIS recovery and NIS correction into account. Figure 1 shows that all target PFAS compounds were taken up and accumulated in plant tissues and could be extracted using the three evaluated methods. In the method blanks and plant control treatment without PFAS exposure (0 ppb), PFAS compounds were not detected, indicating a clean background during extraction and the absence of cross-contamination. The treatment with a higher PFAS exposure concentration (200 ppb) resulted in higher PFAS levels in plant tissues compared to the treatment with a lower exposure concentration (2 ppb). Thus, the treatment concentration (i.e., 2 ppb, 200 ppb) can serve as a proxy for the PFAS levels in plant tissues throughout this paper.

**Figure 1:**
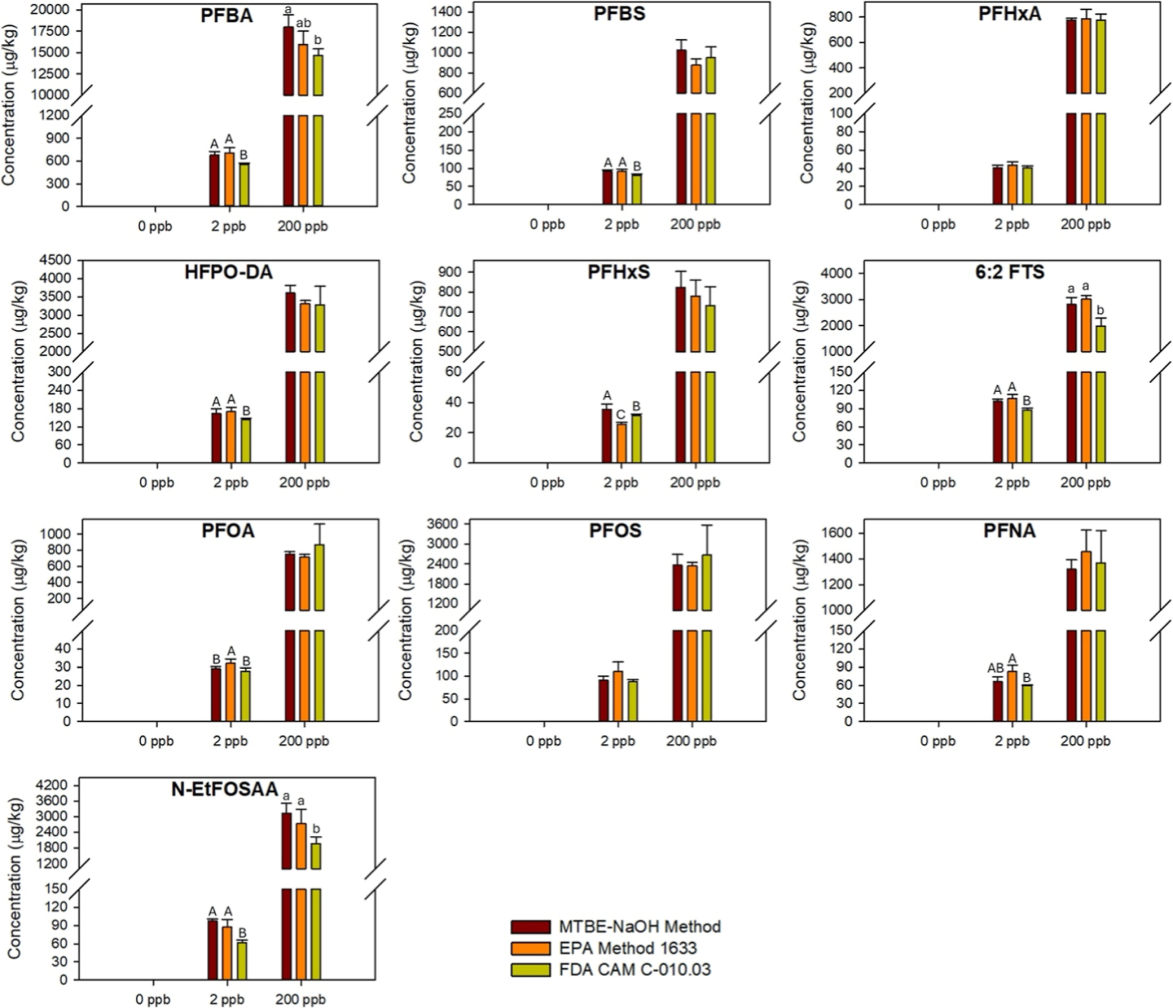
Concentrations of PFAS (μg PFAS/kg dry plant tissues) extracted from soybean plants using three studied methods (*n* = 3). Different uppercase and lowercase letters indicate significant differences among the three extraction methods at the same PFAS exposure concentration (*p* ≤ 0.05).

LLOPR is a QC sample containing twice the lowest PFAS concentration that can be reliably quantified with acceptable precision and accuracy, ensuring the method is sensitive enough for environmentally relevant detection. MLOPR is a QC sample with a midpoint concentration within the PFAS quantitation range and serves as a benchmark for evaluating the method’s routine performance and stability. Ten target PFAS were spiked into the LLOPR and MLOPR samples before extraction to achieve the target concentrations. Comparison of the spiked-PFAS recoveries in LLOPR and MLOPR (Table S6) reveals clear differences among the three extraction methods. MTBE-NaOH method fell within the acceptance ranges for 1 of 10 target PFAS in LLOPR and for 2 of 10 in MLOPR. EPA Method 1633 met 2 of 10 criteria in LLOPR and 3 of 10 in MLOPR. FDA CAM C-010.03 past 3 PFAS in LLOPR and 3 in MLOPR. Overall, all three methods lacked consistent QC performance and compliance.

Table 3 compares the extraction efficiencies for target PFAS in plant tissue samples across the three methods. Generally, in plant tissues with low PFAS levels (2 ppb), EPA method 1633 was the most reliable extraction method, delivering the closest-to-ideal recoveries for six of the target PFAS compounds. However, there were exceptions: for PFBA, PFBS, and *N*-EtFOSAA, the FDA method achieved the best extraction efficiencies, with recoveries closest to 100%. Meanwhile, MTBE-NaOH method outperformed the other two methods for PFHxS. In plant tissues with high PFAS levels (200 ppb), the best-performing method varied by compound. One-way ANOVA with posthoc Tukey tests shows that EPA method 1633 yielded recoveries closest to the 100% target for 6:2 FTS and *N*-EtFOSAA. The FDA method gave the highest mean recovery for PFBA. For the remaining seven target PFAS (PFBS, PFHxA, HFPO–DA, PFHxS, PFOA, PFOS, PFNA), the method effect was not significant (*p* > 0.05).

**Table 3:** Extraction Efficiencies of Target PFAS from Plant Tissue Samples Using the Three Evaluated Methods (*n* = 3)

method	treatment	extraction efficiency (%)									
		PFBA	PFBS	PFHxA	HFPO–DA	PFHxS	6:2 FTS	PFOA	PFOS	PFNA	*N*-EtFOSAA
MTBE-NaOH method	2 ppb	134.69 ± 9.55	114.23 ± 6.36	78.38 ± 5.11	59.62 ± 5.65	35.56 ± 3.44	21.86 ± 0.72	24.07 ± 1.03	69.22 ± 7.17	51.78 ± 5.86	241.09 ± 8.13
	200 ppb	119.56 ± 9.50	72.41 ± 6.93	59.45 ± 1.20	59.74 ± 3.44	25.04 ± 2.43	10.99 ± 1.03	17.99 ± 0.72	25.68 ± 3.46	16.92 ± 0.95	117.99 ± 14.61
EPA method 1633	2 ppb	140.16 ± 14.58	114.47 ± 8.24	84.52 ± 6.41	62.04 ± 4.91	25.88 ± 1.06	22.75 ± 1.48	26.53 ± 2.02	84.41 ± 15.41	64.85 ± 7.82	217.08 ± 31.73
	200 ppb	105.88 ± 10.33	62.16 ± 4.12	60.48 ± 5.43	54.76 ± 1.49	23.72 ± 2.41	11.81 ± 0.48	17.04 ± 0.82	25.42 ± 1.22	18.69 ± 2.16	103.18 ± 20.37
FDA CAM C-010.03	2 ppb	110.92 ± 2.98	101.01 ± 5.31	78.63 ± 3.92	52.11 ± 1.54	31.54 ± 0.96	18.73 ± 0.64	23.01 ± 1.50	67.36 ± 3.37	46.18 ± 0.82	153.99 ± 10.77
	200 ppb	97.55 ± 4.93	67.06 ± 7.67	59.49 ± 3.81	54.29 ± 8.45	22.22 ± 2.93	7.73 ± 1.21	20.70 ± 6.21	28.96 ± 9.82	17.57 ± 3.20	73.99 ± 9.90

Two-way ANOVA was also conducted for extraction efficiency (Table S5), assessing the effects of extraction method and PFAS level in plant tissues (2 ppb vs 200 ppb). The results show that the PFAS level in plant tissues (treatment) was the dominant factor affecting extraction efficiency, significantly influencing 9 out of 10 target PFAS compounds (*p* < 0.05). A higher PFAS level in plant tissues led to suppressed extraction efficiency for these nine compounds. The extraction method was a significant factor for five PFAS compounds (PFBA, PFHxS, 6:2 FTS, PFNA, and *N*-EtFOSAA). A significant method × treatment interaction was observed for PFHxS and PFNA, indicating that the method yielding the highest recovery at 2 ppb was not necessarily the best method at 200 ppb for these two compounds. For the remaining eight target PFAS compounds, the relative performance of the three extraction methods did not vary with PFAS concentration in the plant tissues. Therefore, focusing on extraction efficiency, EPA Method 1633 demonstrated superior performance for 6:2 FTS and *N*-EtFOSAA and was never the worst-performing method, making it the safest single choice. While the MTBE-NaOH and FDA methods provided comparable recoveries for most other compounds, neither surpassed EPA method 1633 in a statistically significant way when plant tissues contained high levels of PFAS.

When both performance criteria, EIS recovery (Table 2) and target PFAS extraction efficiency (Table 3), are considered side by side, the performance of the three evaluated methods showed a consistent PFAS concentration-dependent pattern across the two treatment levels (2 and 200 ppb). For plant tissues with low PFAS levels (2 ppb), MTBE-NaOH method provided the best recovery for five of the ten EIS compounds (M3PFBS, M5PFHxA, M8PFOA, M8PFOS, M9PFNA), while EPA method 1633 delivered the closest-to-100% extraction efficiency for six of the ten target PFAS compounds (PFHxA, HFPO–DA, 6:2 FTS, PFOA, PFOS, PFNA), never ranking last. The FDA method led for three PFAS compounds (PFBA, PFBS, and *N*-EtFOSAA) but only for two EIS compounds (MPFBA, d5-*N*-EtFOSAA). When the PFAS treatment level increased to 200 ppb, the MTBE-NaOH method’s strength in EIS recovery became dominant, achieving the closest-to-100% recoveries for eight of the ten EIS compounds (M3PFBS, M5PFHxA, M3HFPO–DA, M3PFHxS, M8PFOA, M8PFOS, M9PFNA, d5-*N*-EtFOSAA), while the three methods performed similarly in target PFAS extraction efficiency. Overall, EPA Method 1633 remains the safest single method for routine analysis of plant tissues containing environmentally relevant PFAS concentrations, whereas the MTBE-NaOH method becomes the method of choice when accurate isotopic correction based on EIS recovery is critical for highly contaminated plant samples.

It is worth noting that even after applying EIS and NIS isotopic corrections, the recoveries of several target PFAS compounds, including PFHxS, 6:2 FTS, PFOA, PFOS, and PFNA, remained below 50% across all three extraction methods. Moreover, the extraction efficiencies for these compounds further decreased as PFAS levels in plant tissues increased. It suggests that once PFAS compounds are taken up by plants and bioaccumulated in tissues, a portion of them may strongly bind to plant matrices, becoming nonextractable regardless of the extraction method used. Then, isotopically labeled EIS, spiked at a known mass at the beginning of the extraction process, may not be able to fully correct for the recovery of the nonextractable portion. In other words, the binding behavior of the spiked EIS to plant tissues differs from that of PFAS naturally accumulated within the tissues. Given this observation, previous studies investigating PFAS uptake in plants using the extraction methods evaluated in this work may have underestimated the true extent of PFAS accumulation, particularly for the five compounds mentioned above. Further optimization and development of extraction methods involving stronger chemical and/or physical treatments are needed to improve recovery and ensure more accurate assessments of PFAS bioaccumulation in plant tissues.

### 3.3 Cost Evaluation of the Three Extraction Methods

The cost comparison among the three extraction methods demonstrates clear differences in the total consumable cost per sample (Table 4). EPA method 1633 is the most economical, with a total cost of approximately $11.61 per sample. MTBE-NaOH method closely follows, costing around $12.15 per sample, which is only slightly higher than EPA Method 1633. In contrast, FDA CAM C-010.03 is significantly more expensive, nearly doubling the cost of the other two methods at approximately $23.20 per sample. The major cost difference arises from the use of QuEChERS salt ($3.94/packet) and dSPE sorbent ($7.72/packet). Given these cost differences, EPA method 1633 is the most cost-effective option for routine plant-tissue analyses at typical environmental PFAS concentrations, providing an optimal balance between analytical performance and expense. MTBE-NaOH method, despite its slightly higher cost, remains a viable alternative when accurate isotopic correction is crucial, particularly for samples with higher PFAS contamination levels. However, the FDA method, due to its substantially higher cost combined with its relatively lower extraction performance, is not recommended for routine quantitative analyses unless specific analytical requirements justify the higher expenditure.

**Table 4:** Estimated Cost Breakdown for Preparing One Sample Using the Three Evaluated Methods

MTBE-NaOH method	consumable list	quantity	unit price	total cost
	50 mL centrifuge tube	2	0.69	$1.37
	15 mL centrifuge tube	1	0.53	$0.53
	1.5 mL microcentrifuge tube	1	0.06	$0.06
	MTBE (mL)	15	0.13	$1.88
	NaOH (mL)	5	0.00	$0.02
	TBAHS (mL)	2	0.11	$0.21
	Na2CO3 (mL)	5	0.00	$0.01
	methanol (mL)	10	0.05	$0.46
	LCMS vials	1	0.46	$0.46
	bond Elut WAX Cartridge	1	6.90	$6.90
	formic acid (ul)	120	2.03	$0.24
	ammonium hydroxide (μL)	10	0.16	$0.01
	cost per sample			$12.15
EPA method 1633	50 mL centrifuge tube	2	0.69	$1.37
	15 mL centrifuge tube	1	0.53	$0.53
	1.5 mL microcentrifuge tube	1	0.06	$0.06
	methanol (mL)	20	0.05	$0.92
	acetonitrile (mL)	10	0.11	$1.10
	supelclean envicarb (mg)	10	0.19	$0.01
	formic acid (uL)	120	2.03	$0.24
	ammonium hydroxide (μL)	10	0.16	$0.01
	LCMS vials	1	0.46	$0.46
	bond Elut WAX Cartridge	1	6.90	$6.90
	cost per sample			$11.61
FDA CAM C-010.03	50 mL centrifuge tube	2	0.69	$1.37
	15 mL centrifuge tube	2	0.53	$1.05
	1.5 mL microcentrifuge tube	1	0.06	$0.06
	methanol (ml)	5	0.05	$0.23
	formic acid (μL)	170	2.03	$0.35
	acetonitrile (ml)	10	0.11	$1.10
	QuEChERS salt packet	1	3.94	$3.94
	dSPE sorbent	1	7.72	$7.72
	ammonium hydroxide (μL)	130	0.16	$0.02
	LCMS vials	1	0.46	$0.46
	bond Elut WAX Cartridge	1	6.90	$6.90
	cost per sample			$23.20

### 3.4 Research Implications

This research work demonstrated that EPA Method 1633 consistently delivered the highest and most reproducible EIS recoveries at environmentally relevant low PFAS concentrations, while MTBE-NaOH method excelled in EIS recovery at elevated concentrations, despite showing greater variability. For target PFAS extraction efficiency, EPA method 1633 emerged as the most reliable choice for plant tissues containing low PFAS concentrations. MTBE-NaOH method provided comparable performance for select compounds but was optimal primarily for highly contaminated plant samples. A cost comparison further supported adopting EPA method 1633 as the default method for routine plant-tissue analyses at typical environmental concentrations, supplemented by MTBE-NaOH when precise isotopic correction is critical for highly contaminated samples. The results also highlight the differences in the binding behavior between spiked EIS and naturally accumulated PFAS compounds within plant tissues. Consistently low recoveries for certain strongly bound PFAS indicate the need for further method optimization. Future studies should explore enhanced extraction techniques to more accurately quantify these compounds, ensuring robust assessments of PFAS bioaccumulation in agricultural and food safety contexts. Moreover, further studies are needed to assess the adaptability of the evaluated extraction methods to different crop types and their potential environmental applications.

## Supplementary Material



## Notes

Detailed descriptions of the materials used in this study, additional instrument setup information, and supplementary PFAS recovery data with associated statistical analyses (PDF)
