# Supplementary material for: Evaluation and Comparison of Three Common Methods for PFAS Extraction from Soybean Tissues
Source: ACS Agric Sci Technol. 2025 Nov 13;6(3):434–42. doi: 10.1021/acsagscitech.5c00518 (PMC12997148; doi:10.1021/acsagscitech.5c00518)
Supplement: Supplementary file 1 [file as5c00518_si_001.pdf]

Evaluation and Comparison of Three Common Methods for PFAS Extraction from Soybean Tissues

Madhav Kharel [#], Yuwei Zuo [#], Weilan Zhang [*]

Department of Environmental & Sustainable Engineering, University at Albany, State University of New York, Albany, NY 12222, United States

[#] These authors contributed equally to this work.

[*] Corresponding author: Weilan Zhang

Email: wzhang4@albany.edu

Address: 1400 Washington Avenue, Albany, NY 12222, USA.

Table S1. Chemicals, reagents, and consumables used in this study.

Chemical name	Grade/purity	Supplier
Supelclean Envicarb	N/A	Supelco, Inc
Hexafluoropropylene oxide dimer acid (HFPO-DA)	≥98%	Fisher Scientific
Perfluorohexanoic acid	≥98%	Frontier Scientific
Perfluorobutanoic acid	≥97%	Fisher scientific
Perfluorooctanoic acid (PFOA)	≥96%	Sigma-Aldrich
Perfluorononanoic acid (PFNA)	≥98%	Oakwood chemicals
Potassium perfluorobutanesulfonate(PFBS)	≥98%	Fisher scientific
Perfluorohexanesulfonic acid potassium salt (PFHxS)	≥98%	Fisher Scientific
Perfluorooctanesulfonic acid (PFOS)	≥98%	Fisher scientific
6:2 Fluorotelomer sulfonic acid	≥98%	Laboratories SynQuest
2-N-Ethyl(perfluorooctanesulfonamido)acetic acid	≥95%	Laboratories SynQuest
Perfluoro-n-[1,2-13C2]octanoic acid	≥98%	Wellington Laboratories Inc.
Sodium perfluoro-1[1,2,3,4-^{13}C4]octanesulfonate	≥98	Wellington Laboratories Inc.
Perfluoro-n-[1,2,3,4,6-^{13}C5]hexanoic acid	≥98%	Wellington Laboratories Inc.
Ammonium acetate	LCMS grade	Fisher Scientific
Methanol	LCMS/HPLC grade	Fisher Scientific
Ammonium hydroxide	28-30 %	Fisher Scientific
Sodium hydroxide	Certified ACS	Fisher Scientific
Acetic Acid	≥99%	Fisher Scientific
Water	LCMS Grade	Fisher Scientific
Ottawa Sand	Certified ACS	Fisher Scientific
Methyl tert-butyl ether	Certified ACS	Fisher Scientific
Sodium Carbonate	≥98%	Thermo Scientific
Tetrabutylammonium hydrogensulfate		Tokyo Chemical Industry Co. Ltd.
Formic Acid	Certified ACS	Fisher Scientific
Acetonitrile	Certified ACS	Fisher Scientific
Methanol	LCMS grade	Sigma Aldrich
Potassium Hydroxide	Certified ACS	Fisher Scientific
Sodium sulfate Anhydrous	Certified ACS	Fisher Scientific
Sulphuric Acid	Certified ACS	Supelco, Inc
Glacial Acetic Acid	Certified ACS	Fisher Scientific
PP vials and caps for LC-MS/MS analysis	N/A	Thermo Scientific

Hoagland basal salt mixture	N/A	Phytotechnology Laboratories
Q-sep QuEChERS dSPE	N/A	Restek
Q-sep QuEChERS dSPE Extraction Packets	N/A	Restek
Soybean seeds	N/A	Johnny's Selected Seeds

Table S2: Ingredients of Hoagland Modified Basal Salt Mixture.

**Ingredients**	**CAS Number**	**%**
Ammonium Phosphate, Monobasic	77222-76-1	7.05
Potassium Nitrate	7757-79-1	37.22
Calcium Nitrate	13477-34-4	40.27
EDTA, Disodium salt, Dihydrate	6381-92-6	0.22
Ferrous Sulfate·7H2O	7782-63-0	0.15
Cupric Sulfate·5H2O	7758-99-8	0.005
Molybdenum Trioxide	1313-27-5	0.001
Manganese Chloride·4H2O	13446-34-9	0.11
Boric Acid	10043-35-3	0.18

Table S3. Details of the LC-MS/MS instrument setup, limits of detection (LOD) and quantification (LOQ) for each PFAS compound, and R^2 values of the PFAS calibration curves.

LC-MS/MS Conditions	
**Parameter**	**Description**
Column	Agilent ZORBAX Eclipse Plus C18 (3.0 × 50 mm, 1.8 µm)
Mobile Phases	A: 5mM ammonium acetate in 100% water;<br>B: 5mM ammonium acetate in 95% methanol and 5% water
Gradient Elution	0 - 8 min: 70% A and 30% B<br>8 - 10.5 min: 0% A and 100% B
Flow Rate	0.5 mL/min
Column Temperature	50 °C
Injection Volume	5 µL
Run Time	10.50 min

Analyte	LOD (ng/L)	LOQ (ng/L)	R^2
PFBA	78.125	156.25	0.9996
PFBS	9.765	19.531	0.9997
PFHxA	19.531	39.063	0.9998
HFPO-DA	78.125	156.25	0.9991
PFHxS	19.531	39.063	0.9990
6:2 FTS	39.063	78.125	0.9991
PFOA	39.063	78.125	0.9994
PFOS	19.531	39.063	0.9985
PFNA	9.765	19.531	0.9998
NEtFOSAA	39.063	78.125	0.9970

Table S4. EIS recoveries in quality control samples using the methods evaluated and acceptance limits for EIS recoveries in tissue samples specified in EPA Method 1633.

Method	QC sample	Extracted Internal Standard Recovery (%)									
		**MPFBA**	**M3PFBS**	**M5PFHxA**	**M3HFPO-DA**	**M3PFHxS**	**M2-6:2 FTS**	**M8PFOA**	**M8PFOS**	**M9PFNA**	**d5-N-EtFOSAA**
**MTBE-NaOH Method**	Method blank	36.91	75.74	70.32	60.32	74.27	106.29	66.21	67.46	70.23	92.66
	LLOPR	37.43	76.20	71.00	59.71	74.51	112.94	70.71	67.85	68.51	92.31
	MLOPR	38.39	72.03	62.92	56.65	70.52	102.32	60.52	65.42	59.88	81.01
**EPA Method 1633**	Method blank	47.61	49.39	48.51	46.76	50.71	77.15	45.63	47.27	44.11	54.39
	LLOPR	42.72	47.07	44.53	44.12	50.25	65.96	45.03	43.25	45.34	47.32
	MLOPR	48.37	51.16	48.00	51.03	52.84	75.26	46.85	45.42	46.56	46.78
**FDA CAM C-010.03**	Method blank	55.36	59.35	55.64	54.81	55.95	90.66	59.41	51.32	58.84	78.57
	LLOPR	50.49	56.15	52.45	51.87	54.18	75.87	57.29	49.52	54.10	63.89
	MLOPR	52.88	58.10	56.62	52.06	55.44	99.36	56.54	47.63	56.48	64.38
**EPA Method 1633 EIS Recovery Acceptance Limits**		**MPFBA**	**M3PFBS**	**M5PFHxA**	**M3HFPO-DA**	**M3PFHxS**	**M2-6:2 FTS**	**M8PFOA**	**M8PFOS**	**M9PFNA**	**d5-N-EtFOSAA**
		5 - 130	25 - 190	25 - 170	20 - 185	35 - 175	35 - 300	25 - 150	40 - 160	35 - 185	30 - 235

Table S5. Two-way ANOVA for EIS recovery and extraction efficiency among three methods (n = 3).

**Source**	**EIS Recovery Two-Way ANOVA**				**Extraction Efficiency Two-Way ANOVA**			
	Variable	df	F	p-value	Variable	df	F	p-value
Method (Extraction method)	MPFBA	2	21.876	0.203	PFBA	2	75.739	0.003
	M3PFBS	2	41.832	0.001	PFBS	2	16.613	0.089
	M5PFHxA	2	37.713	0.026	PFHxA	2	32.482	0.347
	M3HFPODA	2	36.222	0.149	HFPO-DA	2	11.098	0.091
	M3PFHxS	2	41.510	0.009	PFHxS	2	17.333	0.006
	M262FTS	2	29.275	0.000	6:2FTS	2	10.934	0.000
	M8PFOA	2	29.285	0.000	PFOA	2	9.455	0.854
	M8PFOS	2	34.106	0.000	PFOS	2	9.153	0.264
	M9PFNA	2	18.276	0.000	PFNA	2	12.371	0.005
	d5NEtFOSAA	2	5.980	0.131	N-EtFOSAA	2	3.982	0.000
Treatment (PFAS exposure concentration)	MPFBA	2	3.459	0.001	PFBA	1	72.766	0.001
	M3PFBS	2	1.556	0.569	PFBS	1	269.912	0.000
	M5PFHxA	2	5.094	0.001	PFHxA	1	146.549	0.000
	M3HFPODA	2	4.156	0.002	HFPO-DA	1	4.823	0.486
	M3PFHxS	2	2.771	0.082	PFHxS	1	91.670	0.000
	M262FTS	2	64.121	0.000	6:2FTS	1	713.244	0.000
	M8PFOA	2	4.517	0.007	PFOA	1	23.220	0.001
	M8PFOS	2	5.661	0.002	PFOS	1	244.687	0.000
	M9PFNA	2	4.990	0.007	PFNA	1	609.788	0.000
	d5NEtFOSAA	2	14.356	0.417	N-EtFOSAA	1	353.594	0.000
Method × Treatment	MPFBA	4	1.795	0.043	PFBA	2	6.424	0.146
	M3PFBS	4	1.271	0.658	PFBS	2	3.596	0.092
	M5PFHxA	4	1.611	0.037	PFHxA	2	1.779	0.571
	M3HFPODA	4	3.650	0.176	HFPO-DA	2	4.401	0.252
	M3PFHxS	4	3.358	0.132	PFHxS	2	4.437	0.022
	M262FTS	4	6.449	0.362	6:2FTS	2	0.285	0.993
	M8PFOA	4	2.636	0.760	PFOA	2	2.827	0.128
	M8PFOS	4	2.286	0.983	PFOS	2	1.656	0.122
	M9PFNA	4	2.078	0.951	PFNA	2	3.008	0.013
	d5NEtFOSAA	4	5.772	0.238	N-EtFOSAA	2	4.128	0.131

Table S6. Extraction efficiencies of target PFAS in ongoing precision and recovery standards (OPR) samples and acceptance limits for target PFAS in OPR are specified in EPA Method 1633.

Method	QC sample	Extraction Efficiency (%)									
		**PFBA**	**PFBS**	**PFHxA**	**HFPO-DA**	**PFHxS**	**6:2 FTS**	**PFOA**	**PFOS**	**PFNA**	**N-EtFOSAA**
**MTBE-NaOH Method**	LLOPR	304.26	41.30	43.16	180.51	38.80	163.28	40.23	60.14	46.45	50.52
	MLOPR	150.94	29.92	29.15	127.28	29.77	127.29	28.85	36.87	34.15	37.35
**EPA Method 1633**	LLOPR	145.65	37.01	38.58	137.86	29.87	137.76	27.32	41.33	37.29	28.02
	MLOPR	112.91	27.59	27.54	107.19	26.45	113.63	26.45	31.30	27.83	26.61
**FDA CAM C-010.03**	LLOPR	123.26	31.02	32.76	117.28	27.70	119.77	21.47	36.10	31.25	20.75
	MLOPR	103.28	24.29	23.35	105.06	25.21	86.08	21.92	29.85	22.94	19.34
**EPA Method 1633 Target PFAS Recovery (%) Acceptance Limits in OPR**		**PFBA**	**PFBS**	**PFHxA**	**HFPO-DA**	**PFHxS**	**6:2 FTS**	**PFOA**	**PFOS**	**PFNA**	**N-EtFOSAA**
		60-140	60-150	60-160	60-145	60-155	60-150	60-150	60-160	60-145	60-145
